# Photoinduced, reversible phase transitions in all-inorganic perovskite nanocrystals

**DOI:** 10.1038/s41467-019-08362-3

**Published:** 2019-01-30

**Authors:** Matthew S. Kirschner, Benjamin T. Diroll, Peijun Guo, Samantha M. Harvey, Waleed Helweh, Nathan C. Flanders, Alexandra Brumberg, Nicolas E. Watkins, Ariel A. Leonard, Austin M. Evans, Michael R. Wasielewski, William R. Dichtel, Xiaoyi Zhang, Lin X. Chen, Richard D. Schaller

**Affiliations:** 1 Department of Chemistry, Northwestern University, Evanston, IL 60208 USA; 2 Center for Nanoscale Materials, Argonne National Laboratory, Lemont, IL 60439 USA; 3 Chemical Science and Engineering, Argonne National Laboratory, Lemont, IL 60439 USA; 4 X-ray Science Division, Argonne National Laboratory, Lemont, IL 60439 USA

## Abstract

Significant interest exists in lead trihalides that present the perovskite structure owing to their demonstrated potential in photovoltaic, lasing, and display applications. These materials are also notable for their unusual phase behavior often displaying easily accessible phase transitions. In this work, time-resolved X-ray diffraction, performed on perovskite cesium lead bromide nanocrystals, maps the lattice response to controlled excitation fluence. These nanocrystals undergo a reversible, photoinduced orthorhombic-to-cubic phase transition which is discernible at fluences greater than 0.34 mJ cm^−2^ through the loss of orthorhombic features and shifting of high-symmetry peaks. This transition recovers on the timescale of 510 ± 100 ps. A reversible crystalline-to-amorphous transition, observable through loss of Bragg diffraction intensity, occurs at higher fluences (greater than 2.5 mJ cm^−2^). These results demonstrate that light-driven phase transitions occur in perovskite materials, which will impact optoelectronic applications and enable the manipulation of non-equilibrium phase characteristics of the broad perovskite material class.

## Abstract (web summary)

Lead trihalide perovskites are notable for their excellent optoelectronic properties and uncommon phase behavior. Here, Kirschner et al. show that cesium lead bromide nanocrystals experience a reversible orthorhombic-to-cubic phase transition at moderate excitation fluences and become amorphous at higher fluences.

## Introduction

Much of the work on lead halide perovskites has focused on understanding the origin of their impressive optoelectronic properties including a tunable bandgap, high carrier mobility, long carrier lifetimes, and large absorption cross section^1–8^. Such advances have enabled thin film, solution-processable photovoltaics with efficiencies comparable to state of the art silicon technologies^1,9–14^ as well as low-threshold lasers^15,16^ and highly efficient light emitting diodes^17–19^. However, the effects of the heating induced from the injection conditions relevant to display and gain applications are not yet understood. Among the possible responses are crystal phase transitions^20–24^ that could become accessible for even moderate excitation fluences. These materials exhibit poor thermal conductivity^25^ which is compounded in nanocrystal (NC) systems which have low interfacial thermal conductance^26^. Improving our understanding of these phenomena may also provide insight into perovskite phase stability that will be important in applications^20,27,28^. Previous investigations of lead halides have demonstrated slow thermalization times in hybrid perovskites^29–34^, partially attributable to the mismatch between the phonon density of states for the organic and inorganic sub-lattices. Studies on the response of nanocrystals to high fluence excitations have revealed fast biexciton lifetimes^4,35^, although the effects of Auger heating on the integrity of the NC lattice remain unexplored. This concern is particularly relevant as elevated temperatures have been shown to reduce photoluminescence, although resiliency can be improved by tuning the halide composition^36^.

One methodology for examining the implications of photoinduced heating in perovskites is time-resolved X-ray diffraction (TR-XRD). Through exciting above the bandgap, fast electron–phonon coupling and Auger heating impulsively deposit energy into the lattice, which could initiate a phase transition. Examining this effect in perovskite NCs will help evaluate their stability under the high carrier injection conditions that they will experience in display and lasing applications. In the past, TR-XRD has enabled characterization of a reversible crystalline-to-amorphous transition (melting) in cadmium selenide NCs^37^ which decreases device performance under intense excitation^38^. Here, we perform TR-XRD on cesium lead bromide NCs, which are prototypical all-inorganic lead trihalide perovskite NCs^39–42^. The studies reveal multiple regimes of material response ranging from a reversible orthorhombic-to-cubic phase transition, up to reversible, and then irreversible melting. In addition to characterizing these photoinduced phase transitions, this work demonstrates that TR-XRD is a promising methodology for understanding phase transitions in perovskite materials.

## Results

### Phases of CsPbBr_3_

**Fig. 1:**
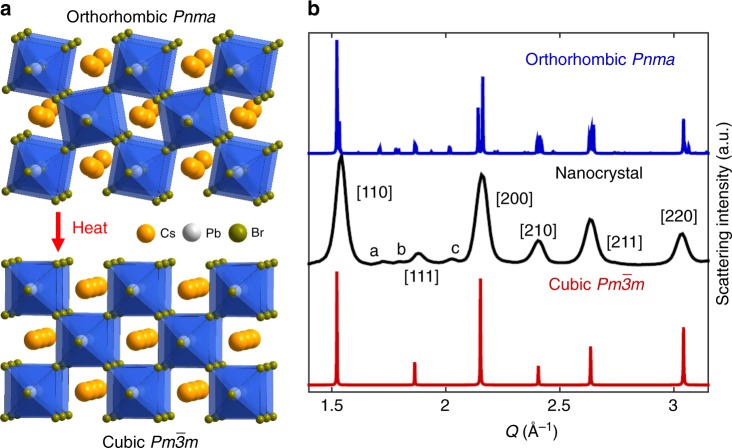
Comparison of orthorhombic and cubic phases. **a** Schematic of CsPbBr_3_ in the orthorhombic (top) and cubic phases (bottom). **b** XRD patterns for the orthorhombic (blue) and cubic phases (red) as generated with VESTA along with the experimental NC XRD pattern (black). The bulk crystal structures are based on a CIF data from Stoumpos et al.^22^ which were adapted with permission. Copyright 2013 American Chemical Society

Figure 1a shows crystal structures of CsPbBr_3_ in the orthorhombic and higher-temperature (above 130 °C in the bulk and 117 °C in the NCs) cubic phases that are differentiated by PbBr_6_ octahedral tilting in the orthorhombic phase as have previously been characterized^20–24^. The resulting reduction in symmetry has implications in the XRD patterns as demonstrated in Fig. 1b. Primarily, the orthorhombic phase (blue) has a much higher density of diffraction peaks than the cubic phase (red), most of which are located near corresponding cubic features. For NCs, Scherrer broadening smears together these closely-spaced peaks such that a single broader peak appears relative to the expected width in the cubic phase. There are also several diffraction peaks absent from the cubic phase. The clearest of these features occur around *Q* = 1.7, 1.8, and 2 Å^−1^, which are all present in the XRD of CsPbBr_3_ NCs (black) and confirm the orthorhombic structure^43,44^. To emphasize these features, we have labeled them as a, b, and c, respectively, in Fig. 1b. Further, for simplicity we have labeled the high-symmetry peaks—those that occur in both phases—with their corresponding cubic planes in Fig. 1b and will use these assignments to refer to diffraction peaks in the corresponding *Q* region. CsPbBr_3_ can also exist in a tetragonal phase (88 °C < *T* < 130 °C for the bulk material and 59 °C < *T* < 117 °C for the nanocrystals) which has a diffraction pattern very similar to the orthorhombic phase with the notable exception of its lack of the b peak.

### Orthorhombic-to-cubic phase transition

To understand how the NC lattice responds to controlled-fluence, impulsive heating, we performed TR-XRD experiments using Beamline 11-ID-D at the Advanced Photon Source (Argonne National Laboratory). Our general methodology was consistent with previously reported methods^37^. Briefly, a reservoir of 11.2 ± 2.9 nm edge length, oleic acid and oleylamine-passivated CsPbBr_3_ NCs dispersed in dodecane, synthesized according to Protesescu et al.^3^ but scaled by a factor of eight to provide enough sample for experiments, was continuously flowed as a free jet into a dry nitrogen-purged interaction region, which assured measurement of fresh, unperturbed material with each laser pulse. The ability to flow NCs in solution is a practical advantage partially motivating their usage over bulk perovskites for TR-XRD measurements. Pump pulses of 3.1 eV photon energy from a 1.6 ps Ti:sapphire laser with a 10 kHz repetition rate were attenuated and focused to achieve the desired fluence. After a controlled time delay, 11.7 keV X-ray pulses (79 ps fwhm) were directed into the jet, and the resulting 2D diffraction pattern was collected on a time-gated Pilatus 2 M detector and radially integrated. The data was normalized for X-ray flux in the *Q* range of 3.5–3.6 Å^−1^.

**Fig. 2:**
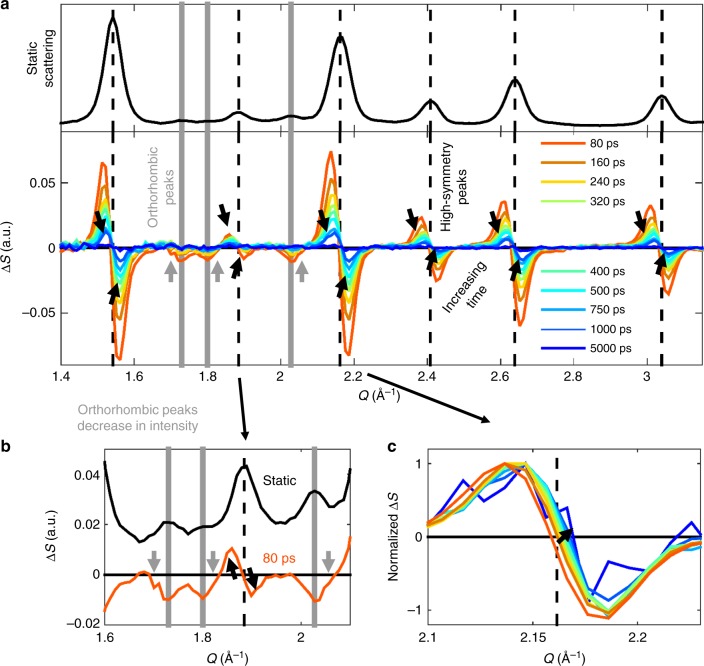
CsPbBr_3_ NC lattice response following moderate photoexcitation. **a** TR-XRD pattern of CsPbBr_3_ NCs at various times following excitation at 4.8 mJ cm^−2^ along with the static XRD pattern for reference on peak positions (black). The orthorhombic peaks are delineated with gray solid lines and the high-symmetry black dashed. A solid black line also denotes Δ*S* = 0 and arrows emphasize how the TR-XRD pattern evolves in time. **b** Zoomed in TR-XRD for 80 ps in the *Q* range of 1.6–2.1 Å^−1^. Once again, the static pattern is displayed with the same features marked as in **a**. Arrows emphasize how the TR-XRD pattern deviates from the static XRD. **c** TR-XRD pattern for the [200] peak normalized such that the maximum Δ*S* = 1. The arrow denotes how the pattern evolves in time

**Fig. 3:**
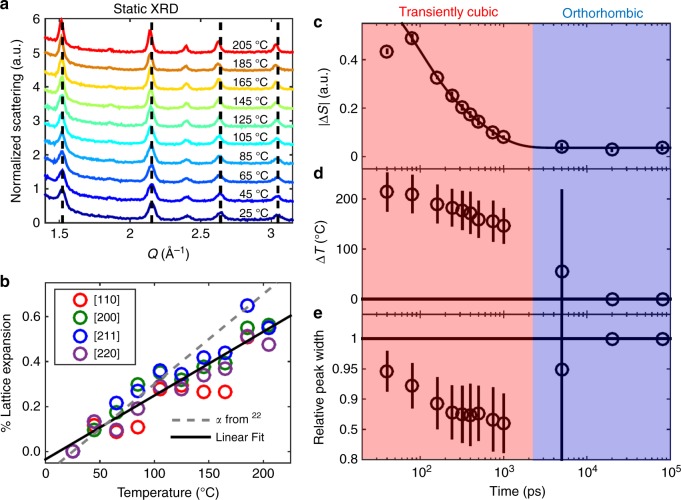
Temporal evolution of NC lattice. **a** Temperature-dependent XRD with black dashed lines to emphasize the room temperature peak position. **b** Expansion of lattice planes from static experiments versus temperature along with a linear fit which suggests a thermal expansion coefficient of 28.4 ± 3.5 × 10^−6^ K^−1^ along with the bulk thermal expansion coefficient from Stoumpos et al.^22^. **c** Integrated absolute change in scattering signal for NCs versus time under a fluence of 4.8 mJ cm^−2^ along with a biexponential fit. Error bars indicate standard deviation in the measurement. **d** Change in NC temperature versus time as calculated from our temperature-dependent XRD measurements. Error bars indicate 95% confidence intervals from our fitting algorithm. **e** Change in relative diffraction peak width (relative to unexcited sample) as calculated from fitting the TR-XRD pattern. Error bars indicate 95% confidence intervals from our fitting algorithm

Figure 2a shows the TR-XRD pattern of CsPbBr_3_ NCs under an excitation fluence of 4.8 mJ cm^–2^. The static XRD pattern is also included in a top panel with vertical lines denoting the orthorhombic (solid, gray) and high-symmetry peak positions (dotted, black). The high-symmetry diffraction features systematically exhibit increased scattering (positive Δ*S*) at lower *Q* and decreased scattering (negative Δ*S*) at higher *Q*, the result of the peaks shifting to lower *Q* values and consistent with thermal expansion. However, the peaks associated with the lower-symmetry orthorhombic phase exhibit distinct behavior, as emphasized in Fig. 2b, which zooms in on the *Q* range of 1.6–2.1 Å^−1^. The TR-XRD signals of these peaks mirror their static XRD patterns with local minima occurring at the static peak positions, a result of a loss of scattering intensity rather than a change in position. Selective reduction in orthorhombic peak intensity suggests that this excitation condition induces a transition to the cubic structure. Related observations have also noted octahedral tilting of lead halides under intense excitation using ultrafast electron diffraction measurements on hybrid perovskite thin films^45^. This photoinduced phase transition is reversible as the orthorhombic peaks completely recover during our observation window and the high-symmetry peaks return to their static positions. This recovery is emphasized in Fig. 2c, which shows the [200] peak normalized at each time as it shifts to the original, higher *Q* position. To convert these shifts in the diffraction peaks to changes in temperature, we collected a series of static, temperature-dependent XRD patterns on a thin film of NCs suspended in a polymer matrix (Fig. 3a). This methodology is used as our experimental data does not follow a Debye–Waller-like dependence as explored in Supplementary Discussion 1 and Supplementary Figure 1. The four most prominent diffraction peaks ([110], [200], [211], and [220] denoted with black dashed lines) were fit to Gaussian functions at each temperature and the shifts in peak positions are plotted in Fig. 3b. Using a linear fit to extract a thermal expansion coefficient yields a value of 28.4 ± 3.5 × 10^−6^ K^−1^ (solid black line) which is on the same order as the measured bulk value of 40 × 10^−6^ K^−1^ (dashed gray line)^22^. The lower value measured for the NCs might be partially caused by annealing, which causes a slight decrease in lattice size after extended times at elevated temperatures (Supplementary Figure 2). Additionally, while the three lattice parameters have been shown to exhibit different temperature dependencies in the bulk, the Scherrer broadening in the NC makes it difficult to distinguish these differences. However, treating the lattice parameters as if they were equivalent still yielded values consistent with bulk values suggesting that this assumption is reasonable. It is also worth noting that the analysis done by Cottingham and Brutchey on refining lattice parameters of temperature-dependent XRD measurements with an assumed cubic structure^24^ would predict a thermal expansion coefficient around 30 × 10^−6^ K^−1^, consistent with our measurements.

Figure 3c shows recovery dynamics of the NC lattice derived from TR-XRD signals from the same four diffraction peaks ([110], [200], [211], [202]), which all exhibit consistent kinetics and fluence dependencies (Supplementary Figures 3 and 4). To account for the fact that the transient signal has negative and positive components, the absolute value of the change in scattering (|Δ*S*|) is used. These kinetics are well fit to a biexponential function with time components of 86 ± 24 ps—possibly due to NC cooling as it is on the order of CdSe NC cooling^46^—and a component of 510 ± 100 ps—which we attribute to the cubic-to-orthorhombic phase transition as it is on the same order as the amorphous-to-crystalline transition in CdSe NCs^37^, the most analogous process. There may also be faster features, which would require higher-time resolution instruments to resolve. We did not observe significant variations in dynamics with different excitation fluences as shown in Supplementary Figure 5. Figure 3d shows NC temperature, calibrated from the thermal expansion coefficient derived from static XRD, as a function of time. While lattice temperature decreases following photoexcitation, the crystals spend an extended amount of time at a plateau around 175 °C. This discontinuity in the change in lattice temperature could be caused by reversion to the orthorhombic phase releasing energy and slowing the cooling process. Additionally, the NCs exhibit narrower peak widths when they are transiently in the cubic phase as displayed in Fig. 3e, likely a result of the increased crystal symmetry as the cubic phase exhibits narrower peaks as shown in Supplementary Figure 6. Some initial cooling causes further peak narrowing for the first nanosecond following photoexcitation until the return to the orthorhombic structure causes broadening to the original peak width.

The mechanism for the deposition of energy into the lattice involves several processes which have already been the subject of thorough investigation. Initially, there is rapid intraband relaxation which has been reported to occur on sub-picosecond timescales^4,35,47^. At higher excitation fluxes, there has been evidence of a hot-phonon bottleneck of a few picoseconds in nanocrystals^35^ that is absent in the bulk measurements^31^. This initial relaxation is understood to be the result of the generation of longitudinal-optical phonons, through Fröhlich electron–phonon coupling, which rapidly downconvert to acoustic phonons^29,30,48^, with the specific vibrational density of states of CsPbBr_3_ having been the subject of previous characterization^30,49^. Additionally, many of the multiexcitons undergo Auger recombination—where one exciton relaxes to the ground state by transferring its energy to another exciton which then proceeds to decay to the band-edge. Generally, biexciton lifetimes depend on NC composition and size—they have been reported to be on the order of around 100 ps for NCs of this size and composition^4,35^—while multiexcitonic rates scale quadratically with number of carriers^50,51^ resulting in rapid recombination under high fluence excitation conditions. Taken together, these methods result in rapid deposition of energy in the lattice. The subsequent phase transition is fundamentally non-equilibrium as it is impulsively induced. The transition pathway is distinct from a thermal transition as it does not proceed through an observable intermediate, tetragonal phase (Supplementary Figure 7) as has been reported in the thermally induced phase transitions of NCs^24^. Similarly, the temperature of the NCs before the cubic-to-orthorhombic transition does not match with the thermal transition which occurs at 117 °C^24^. This non-equilibrium behavior is consistent with the behavior in analogous systems^37,52–54^.

### Fluence dependence

**Fig. 4:**
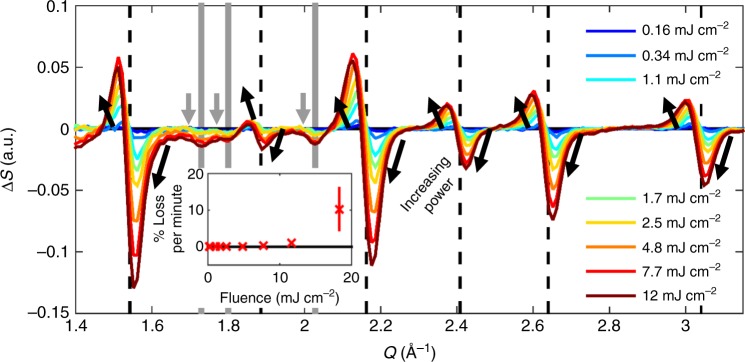
Fluence dependence of lattice deformation. TR-XRD patterns of CsPbBr_3_ NCs 40 ps after photoexcitation at a range of excitation fluences. The orthorhombic peaks are delineated with gray solid lines and the high-symmetry black dashed. A solid black line also denotes Δ*S* = 0 and arrows emphasize how the TR-XRD pattern evolves with power. Inset: Irreversible percent loss of peak intensity versus excitation power. The error bar indicates standard deviation in the measurement

Figure 4 shows the TR-XRD signals 40 ps after photoexcitation at a range of fluences. As the fluence increases, the high-symmetry diffraction peaks move to lower *Q* values resulting in larger TR-XRD signal. However, at the highest fluences, the peaks also decrease in intensity, with the change in scattering signal becoming asymmetric with the negative feature becoming larger in magnitude than the positive feature. This change suggests the initiation of a second phase transition: crystalline-to-amorphous (melting). Additionally, at these higher fluences there was a loss of signal on a laboratory timescale associated with a loss of sample as displayed in the inset. This effect is likely the result of NCs crashing out of solution following the loss of ligands as NCs could be seen deposited on the tubing of our jet. The degradation is negligible at fluences below 7.7 mJ cm^−2^ but at 18 mJ cm^−2^ the degradation was so rapid, extensive, and problematic that we have excluded the corresponding TR-XRD pattern from the main panel. Fluences around 7–12 mJ cm^−2^ should therefore be an upper limit to excitation density for applications of these materials in NC form.

## Discussion

**Fig. 5:**
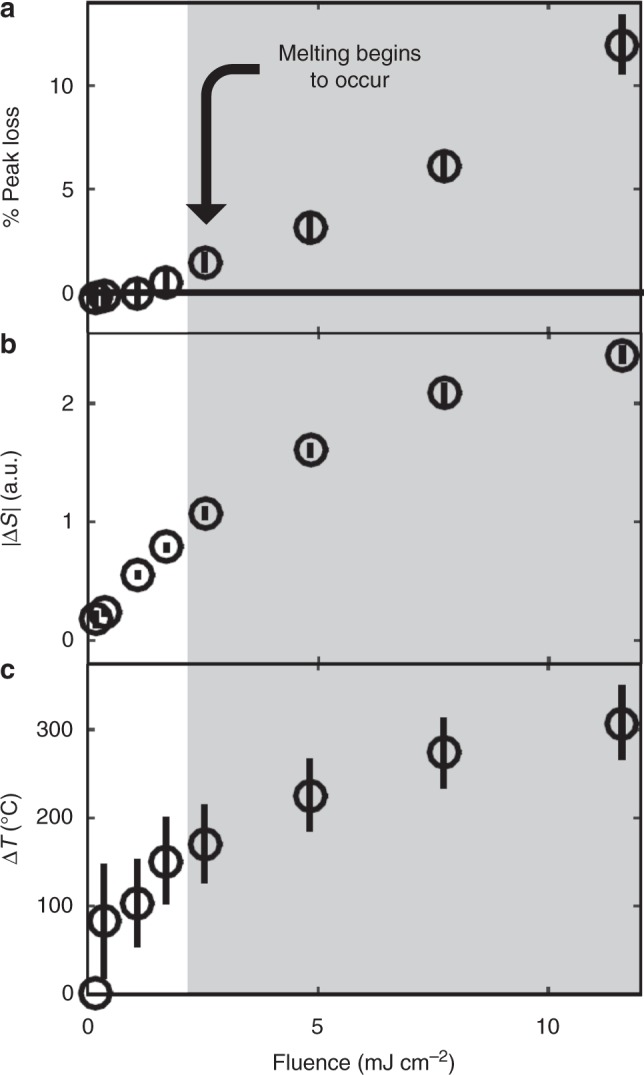
Fluence-dependent lattice temperature and onset of NC melting. **a** Reversible percent loss of peak intensity 40 ps following photoexcitation versus excitation fluence. Error bars indicate standard deviation in the measurement. The gray box indicates fluences past the melting threshold. **b** Integrated absolute change in scattering signal versus excitation fluence. Error bars indicate standard deviation in the measurement. **c** Change in NC temperature versus excitation fluence. Error bars indicate 95% confidence intervals from our fitting algorithm

To determine the onset of melting, we examined the change in total peak intensity as a function of fluence (Fig. 5a). The observed behavior is threshold-like, with no notable changes below the fluence threshold of 2.5 mJ cm^−2^, which is indicative of the start of the crystalline-to-amorphous transition^37^. Figure 5b shows the absolute value of the TR-XRD signal as a function of fluence. This value increases linearly at low fluences but becomes sublinear after the melting threshold. This trend may reflect shifts in peak position contributing more |Δ*S*| than decreases in peak intensity as a result of having both positive and negative components. Percent peak loss and |Δ*S*| exhibit very similar dynamics (Supplementary Figure 8), which is further evidence of the non-equilibrium nature of these phase transitions: instead of transitioning from amorphous to cubic before returning to the orthorhombic phase, the NCs transition directly back to the orthorhombic phase. Figure 5c shows corresponding increases in temperature as calibrated from our temperature-dependent XRD measurements. As shown in Supplementary Figure 9, these measurements also reveal narrower peak widths for NCs in the cubic phase.

In summary, following moderate-to-high-fluence photoexcitation, CsPbBr_3_ NCs experience significant impulsive heating. This heating causes the NCs to undergo an orthorhombic-to-cubic phase transition observable through TR-XRD. Lattice recovery dynamics show extended time at elevated temperatures and narrower diffraction peak widths, providing further evidence of the phase transition which recovers on the timescale of 510 ± 100 ps. At fluences greater than 2.5 mJ cm^−2^, reversible melting begins to occur with significant irreversible damage occurring at fluences greater than 18 mJ cm^−2^. The success of characterizing CsPbBr_3_ with TR-XRD suggests that it is a promising avenue for studying thermal effects in other lead halide perovskites. These processes, particularly phase transitions, need to be considered for perovskite materials under intense excitation, as optoelectronic characteristics have been shown to be strongly influenced by elevated temperatures^36^.

## Methods

### Material synthesis

CsPbBr_3_ NCs, synthesized according to Protesescu et al.^3^ scaled up by a factor of eight. NC size was determined via TEM using a JEOL-1400 microscope.

### Generated X-ray diffraction pattern

The XRD patterns in Fig. 1 were generated using VESTA based on a CIF file from Stoumpos et al.^22^.

### Static X-ray diffraction measurements

Temperature-dependent XRD measurements were performed on a Rigaku Smartlab instrument with a temperature controller under a nitrogen environment.

### Calculating changes in peak position

The changes in peak position were calculated by a global fitting method. First, the static [110], [200], [211], and [202] peaks were fit to Gaussians. Then, at each time point the TR-XRD pattern for each peak *i* was fit to the following equation:

1 $${\mathrm{\Delta }}S_i\left( Q \right) = A_i \left( - {\mathrm{e}}^{ - \left( {\frac{{Q - b_{0{{i}}}}}{{c_{0{{i}}}}}} \right)^2} + \frac{\gamma }{\beta }{\mathrm{e}}^{ - \left( {\frac{{Q - (1 + \alpha )b_{0{{i}}}}}{{\beta c_{0{{i}}}}}} \right)^2}\right)$$

where ${\mathrm{\Delta }}S$ is the change in scattering intensity, *A* is a normalization term, *b*_0_ and c_0_ are the static peak positions, *α* is the lattice expansion, *β* is the relative peak width, and *γ* is a relative peak intensity. This fit represents simply replacing the initial diffraction peak with a shifted peak. While *A* was allowed to vary for each diffraction peak, the other three terms were globally fit to increase the stability of the fitting algorithm.

### Accounting for degradation

To account for degradation, we compared static XRD patterns taken during different scans at the same delay after laser excitation. We used the following equations

2 $$d_{i,j}\left( Q \right) = \frac{1}{n}\mathop {\sum }\limits_{i = 1}^n S_{i,j + 1}\left( Q \right) - S_{i,j}\left( Q \right)$$

3 $$\bar d_j\left( Q \right) = \frac{1}{n}\mathop {\sum }\limits_{i = 1}^n d_{i,j}\left( Q \right)$$

4 $${\mathrm{\Delta }}S_{i,j}\left( Q \right) = S_{i,j}\left( Q \right) - S_{{\tilde m,j}}\left( Q \right) - (i - {\tilde m)} \ast d_j\left( Q \right)$$

where *S*_*i*,*j*_ is scattering intensity for the *i*th time point in the *j*th scan, there are a total of *n* time points in a scan, $\bar d_j\left( Q \right)$ is the average irreversible loss that occurs over scan *j*, and ${\tilde m}$ is the nearest pre-time zero point. For the final scan, we used the value of $\bar d_j\left( Q \right)$ taken from the penultimate scan.

## Supplementary Information

Supplementary Information

## Data Availability

The datasets generated during and/or analyzed during the current study are available from the corresponding author on reasonable request.
