# Supplementary material for: Photoinduced, reversible phase transitions in all-inorganic perovskite nanocrystals
Source: Nat Commun. 2019 Jan 30;10:504. doi: 10.1038/s41467-019-08362-3 (PMC6353988; doi:10.1038/s41467-019-08362-3)
Supplement: Supplementary file 1 — Supplementary Information [file 41467_2019_8362_MOESM1_ESM.pdf]

Supplementary Information for:

Photoinduced, Reversible Phase Transitions in All-Inorganic Perovskite Nanocrystals

Matthew S. Kirschner,[1] Benjamin T. Diroll,[2] Peijun Guo,[2] Samantha M. Harvey,[1] Waleed Helweh,[1] Nathan C. Flanders,[1] Alexandra Brumberg,[1] Nicolas Watkins,[1] Ariel A. Leonard,[1,4] Austin M. Evans,[1] Michael R. Wasielewski,[1] William R. Dichtel,[1] Xiaoyi Zhang,[3] Lin X. Chen,[1,4] Richard D. Schaller[1,2*]

[1]Department of Chemistry, Northwestern University, Evanston, IL 60208

[2]Center for Nanoscale Materials, [3]X-ray Science Division, [4]Chemical Science and Engineering, Argonne National Laboratory, Lemont, IL 60439

*E-mail: schaller@anl.gov; schaller@northwestern.edu

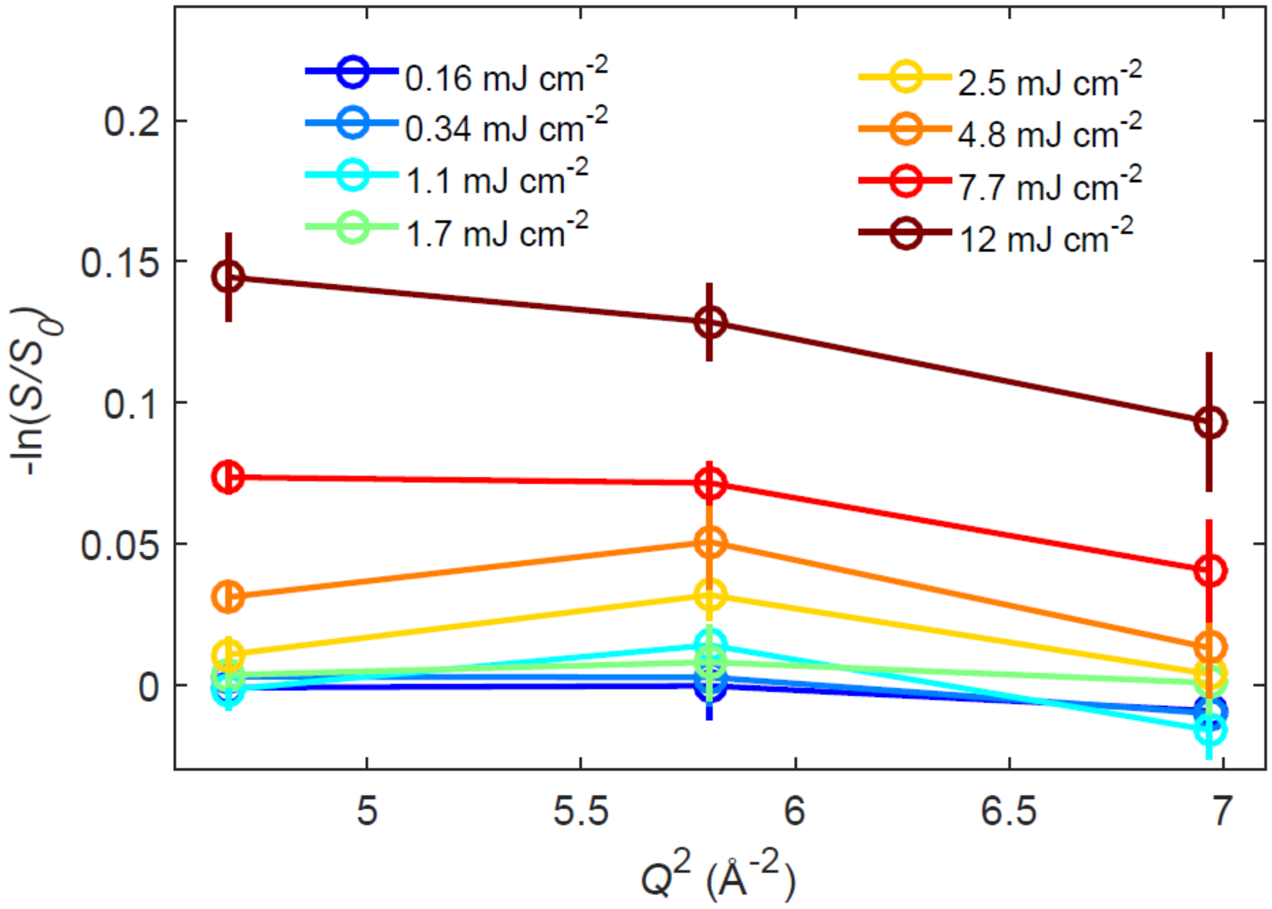

Supplementary Figure 1 | Lack of Debye-Waller Like Dependence. The negative logarithm of scattering intensity 40 ps following photoexcitation over static scattering intensity *vs* Q^2 for a range of photoexcitation fluences. Error bars represent standard deviation in the measurements.

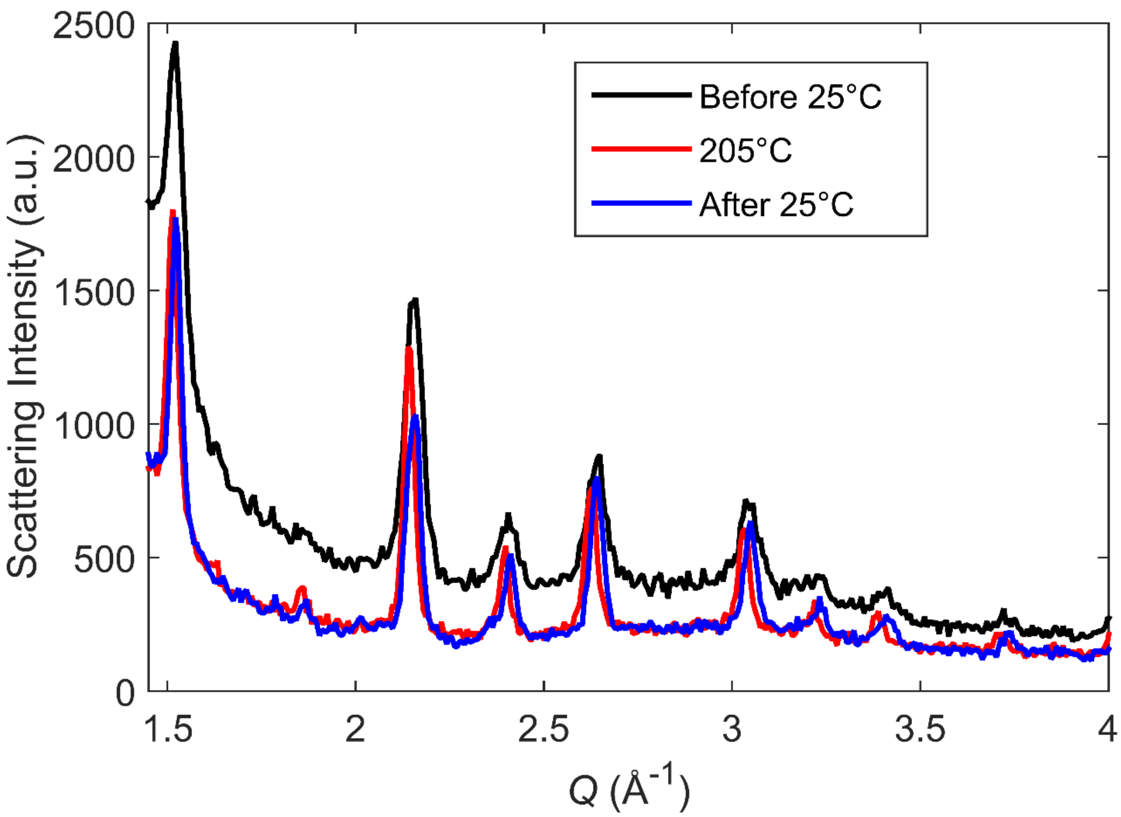

Supplementary Figure 2 | Hysteresis during Temperature Dependent XRD Measurements. XRD measurements of $CsPbBr_3$ NCs before (black) and after (blue) temperature dependent XRD measurements, as well as the XRD pattern at the maximum temperature examined (red).

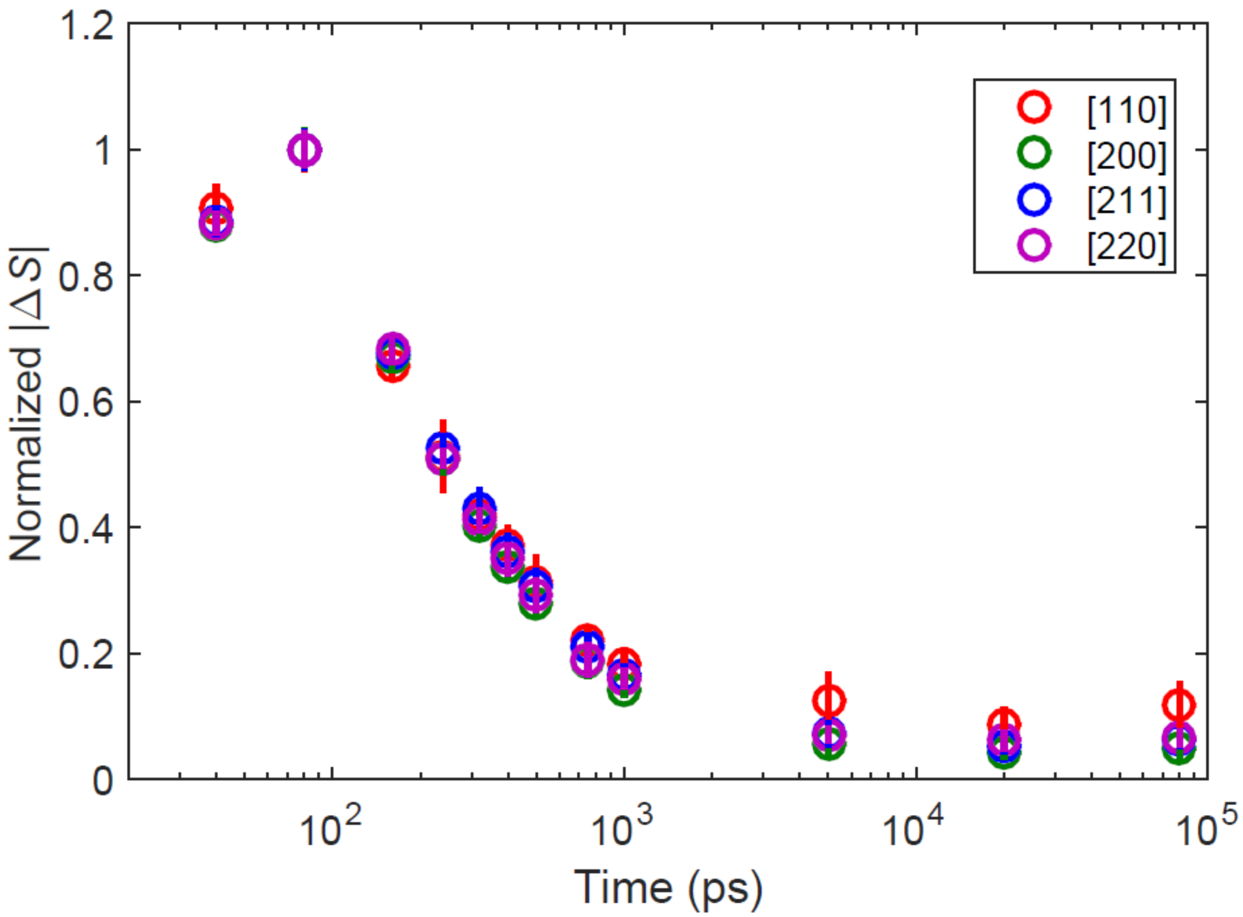

Supplementary Figure 3 | Consistent Recovery Dynamics Across Lattice Planes. Normalized |Δ*S*| for $CsPbBr_3$ NCs following photoexcitation at 4.8 mJ cm^{-2} examined across the four examined diffraction peaks. Error bars represent standard deviations in the measurements.

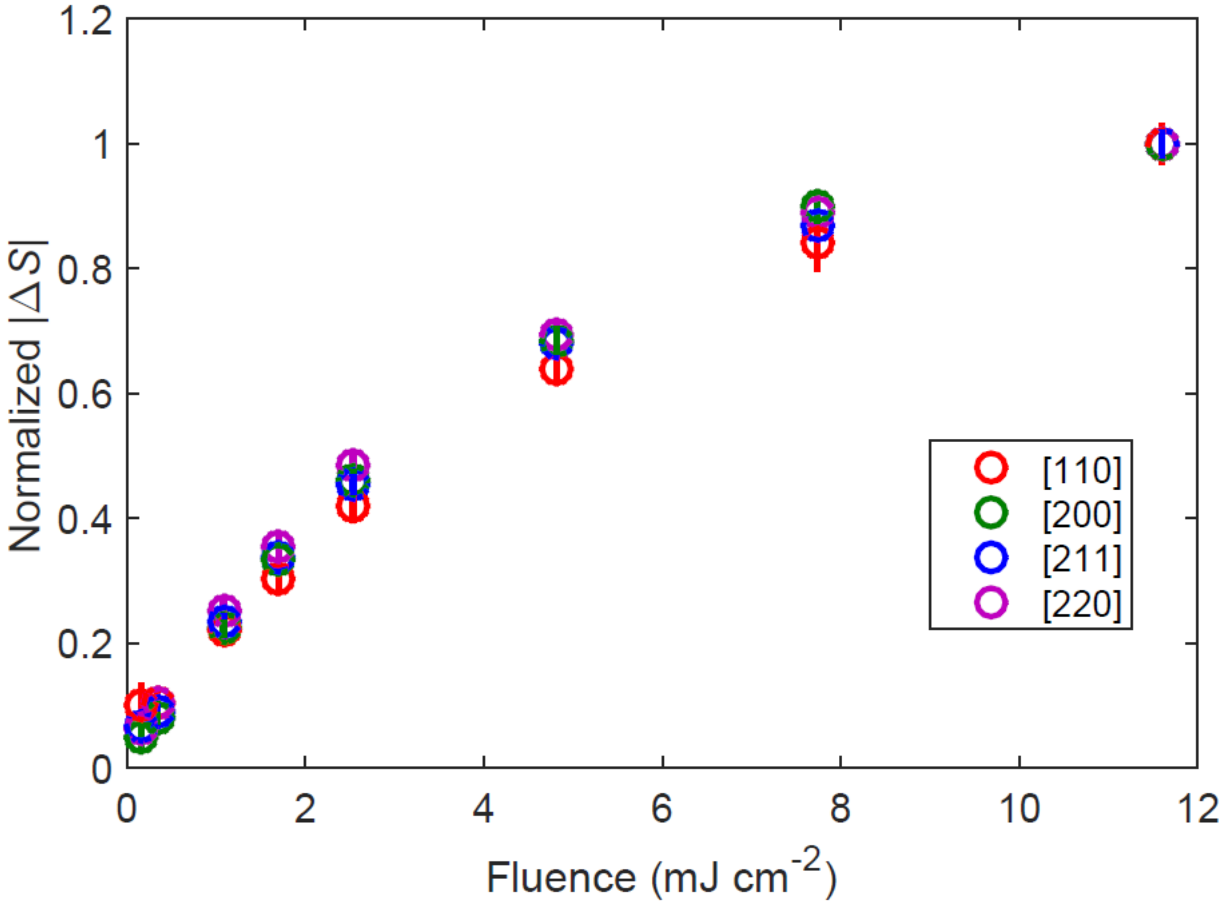

Supplementary Figure 4 | Consistent Fluence Dependence Across Lattice Planes. Normalized |Δ*S*| for $CsPbBr_3$ NCs 40 ps following photoexcitation at a range of fluences examined across the four examined diffraction peaks. Error bars represent standard deviations in the measurements.

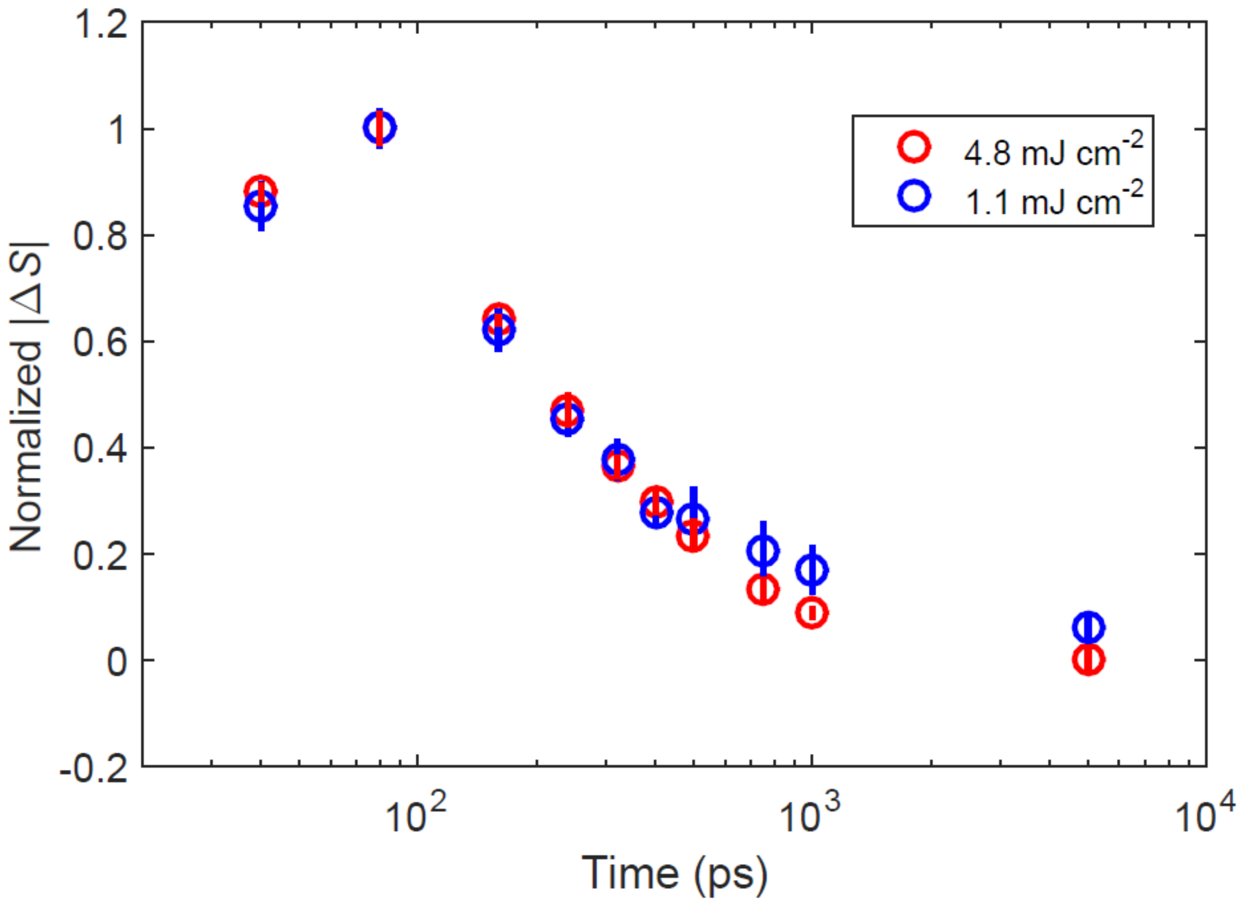

Supplementary Figure 5 | Consistent Recovery Dynamics with Excitation Fluence. Normalized |Δ*S*| for $CsPbBr_3$ NCs following photoexcitation at 1.1 mJ cm^{-2} and 4.8 mJ cm^{-2}. Error bars represent standard deviations in the measurements.

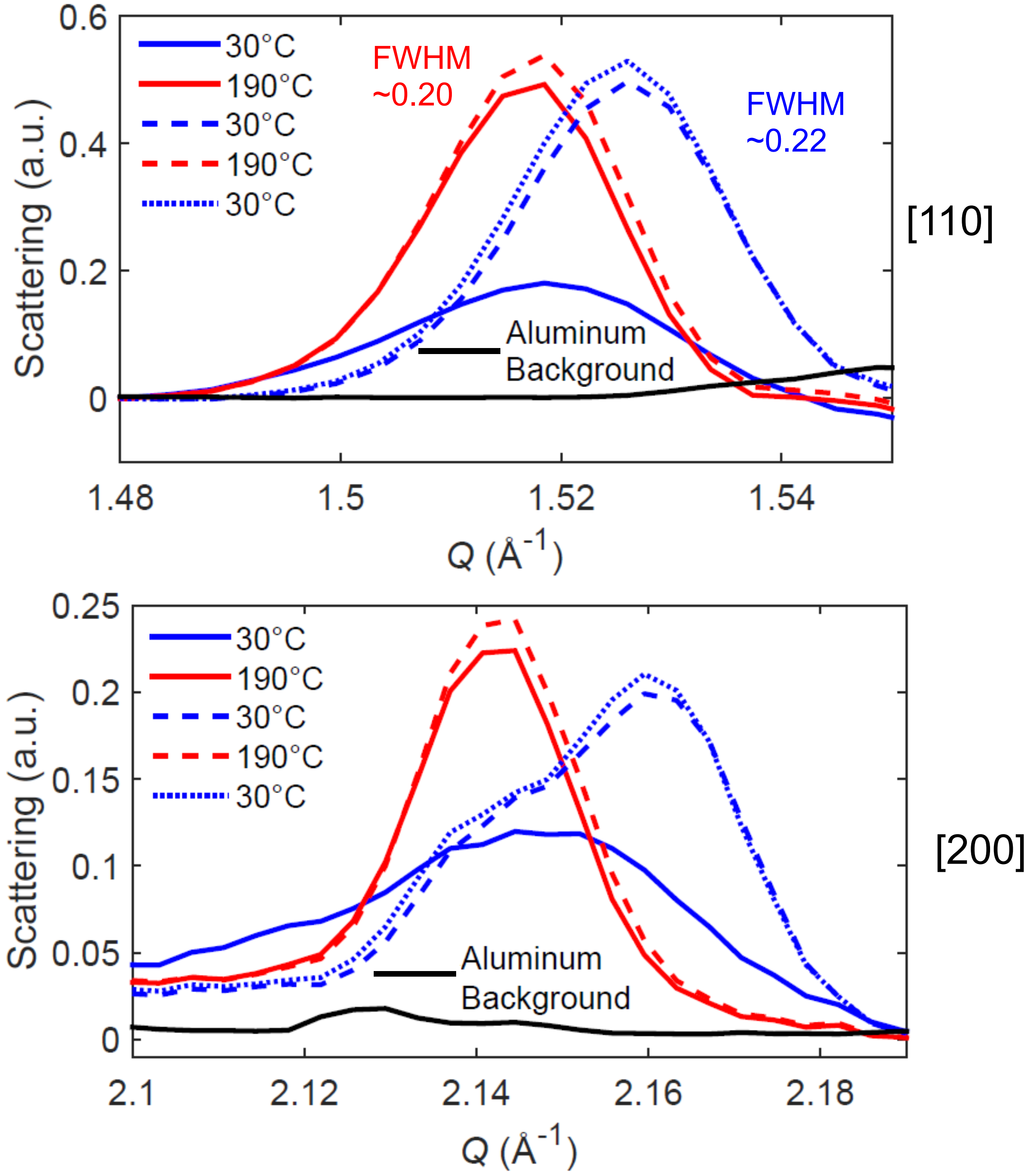

Supplementary Figure 6 | Diffraction Peak Narrowing in the Cubic Phase. Temperature dependent-XRD measurements of the [110] and [200] peaks of $CsPbBr_3$ NCs in a polymer matrix collected at Sector 5 at the Advanced Photon Source. The NCs started at 30°C and then were ramped up to 190°C (solid), back down to 30°C and then ramped up to 190°C (dashed), and then back down to 30°C. While there is an irreversible process occurring during the first increase in temperature, the [110] and [200] peaks are narrower at 190°C where the cubic phase is present. The pattern from the Al crucible is included (black) and was subtracted away from the displayed patterns.

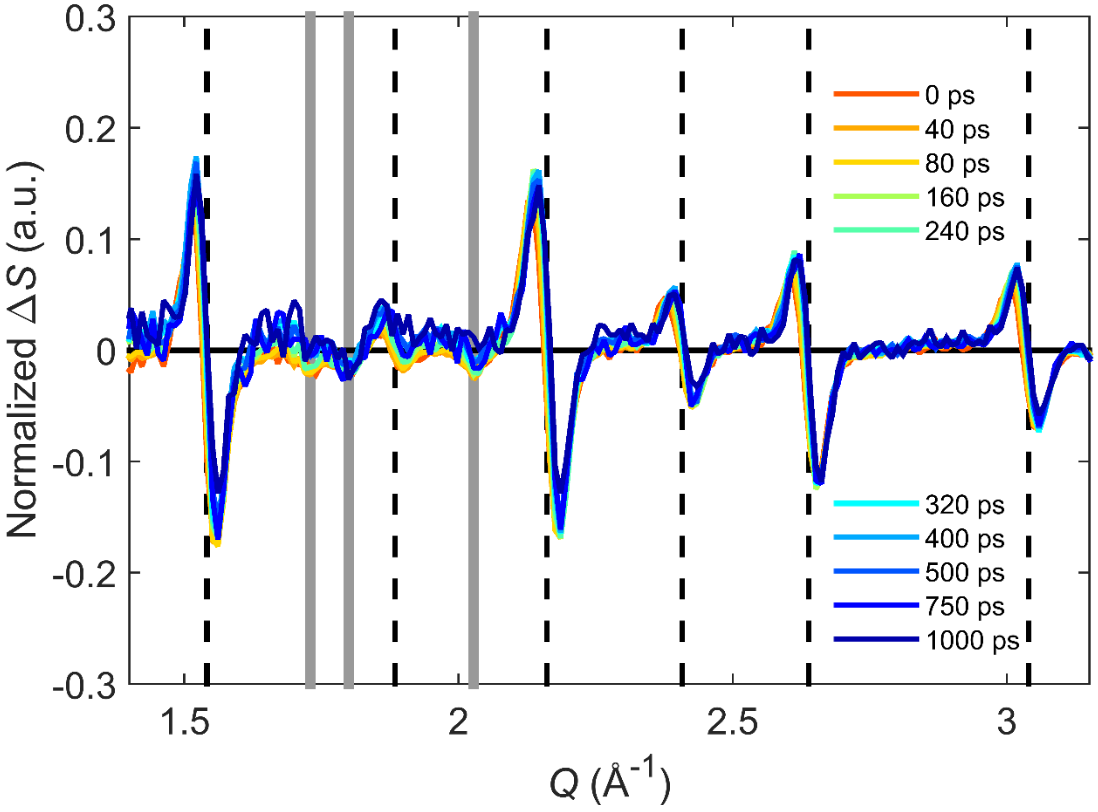

Supplementary Figure 7 | Normalized TR-XRD Patterns Following Photoexcitation. Normalized TR-XRD pattern of CsPbBr3 NCs at various times following excitation at 4.8 mJ cm^{-2}. The orthorhombic peaks are delineated with gray solid lines and the high-symmetry black dashed. A solid black line also denotes Δ*S*=0. The pattern is normalized by dividing by the |Δ*S*|. Notably, the orthorhombic peaks do not change significantly in time.

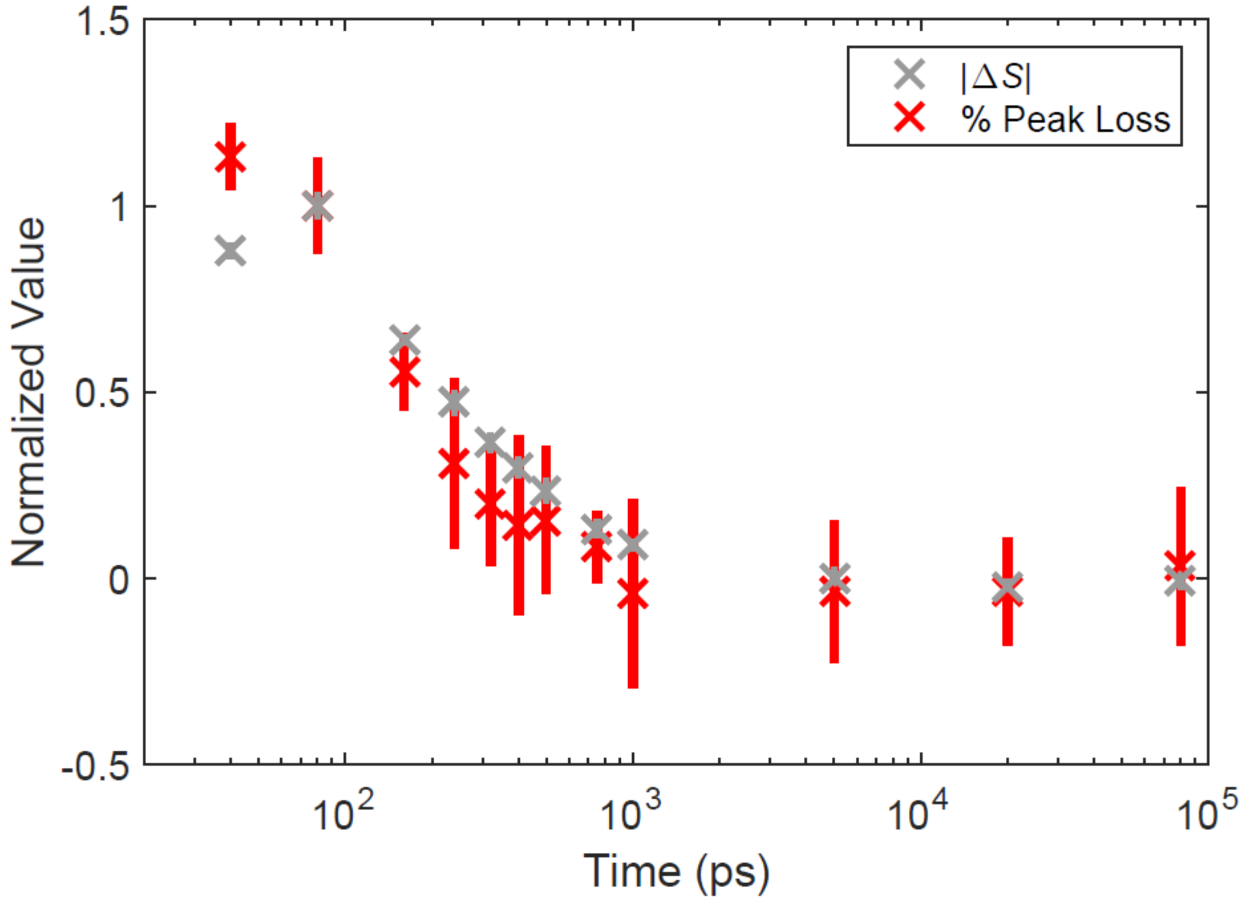

Supplementary Figure 8 | Consistency between |ΔScattering| and Percent Peak Loss. Normalized |Δ*S*| and Percent Peak Loss for $CsPbBr_3$ NCs photoexcited at 4.8 mJ cm^{-2}. Error bars represent standard deviations in the measurements.

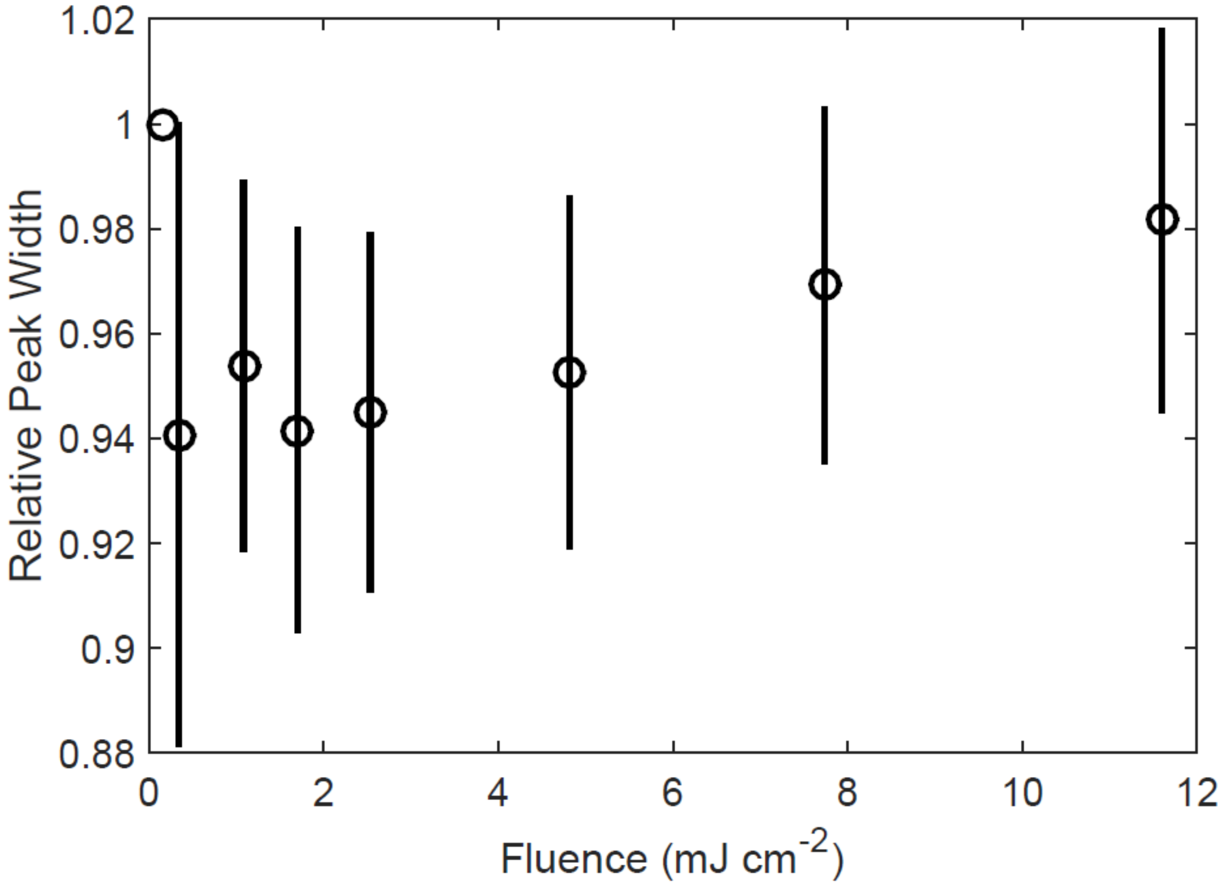

Supplementary Figure 9 | Relative Peak Width vs Fluence. Relative peak width for $CsPbBr_3$ NCs 40 ps following photoexcitation at a range of fluences. Error bars represent 95% confidence intervals in our fitting algorithm.

Supplementary Discussion 1 | Debye Waller Factors.

Measured changes in diffraction patterns do not match a Debye-Waller like dependence, that is $S(Q,t) \propto \exp\left(-\frac{1}{3}Q^2\langle u_{RMS}^2(t)\rangle\right)$ where *S* is the scattering intensity and $\langle u_{RMS}^2(t)\rangle$ is a time-dependent mean square displacement of the atoms[1–3]. If this were the case, we'd be able to see a linear relationship between $-\ln(S/S_0)$ and Q^2 (with S_0 being the static diffraction intensity) which is not observed as shown in Supplementary Figure 1. This may be caused by the phase transition changing the relative intensities of the diffraction peaks.

Supplemental References

1. Lindenberg, A. M. Atomic-Scale Visualization of Inertial Dynamics. *Science (80-.).* **308,** 392–395 (2005).
2. Szilagyi, E. *et al.* Visualization of nanocrystal breathing modes at extreme strains. *Nat. Commun.* **6,** 1–6 (2015).
3. Wu, X. *et al.* Light-induced picosecond rotational disordering of the inorganic sublattice in hybrid perovskites. *Sci. Adv.* e1602388 (2017).
